# Synergistic Effects of Human Platelet-Rich Plasma Combined with Adipose-Derived Stem Cells on Healing in a Mouse Pressure Injury Model

**DOI:** 10.1155/2019/3091619

**Published:** 2019-10-30

**Authors:** Zhiyuan Liu, Shune Xiao, Kerong Tao, Hai Li, Wenhu Jin, Zairong Wei, Dali Wang, Chengliang Deng

**Affiliations:** Department of Plastic Surgery, Affiliated Hospital of Zunyi Medical University, Zunyi, Guizhou, China

## Abstract

Pressure injury (PI) affects quality of life and results in economic and social burdens. Local transplantation of human adipose-derived stem cells (ASCs) is considered an effective treatment. However, ASC suspension alone is vulnerable to the immune system and results in a shortened cell survival. There is increasing evidence of a synergistic effect of platelet-rich plasma (PRP) combined with ASCs on wound healing. This study investigated the effectiveness, synergy, and mechanism of wound healing following local injection of PRP combined with ASCs in a rodent PI model. PRP or ASCs alone were the control intervention. Wound healing, inflammatory infiltration, collagen deposition, angiogenesis, neurogenesis, and cell homing were investigated. PI healing was promoted by the synergistic effects of PRP combined with ASCs. The combination was more effective than ASCs alone for modulating inflammation, increasing collagen deposition, angiogenesis, neurogenesis, and the persistence of the injected ASCs. These data provide a theoretical foundation for the clinical administration of ASCs combined with PRP in PI healing and skin regeneration.

## 1. Introduction

Pressure injury (PI), previously called pressure ulcer, involves loss of the integrity of the epidermis and dermis of the skin, subcutaneous tissue, muscle, and bone caused by continual external force [1]. PI that includes bone erosion may be secondary to infection that can progress to sepsis and be life-threatening. PI occurs in elderly or young patients with paraplegia or quadriplegia because of trauma [2]. Approximately 70% of PIs occur in people older than 70 years of age, and PIs develop in 40% of patients with spinal cord injuries [3, 4]. PIs with delayed healing prolong hospital stays and are prone to recurrence, which increase patient discomfort and have heavy economic and social burdens. The estimated cost of preventing PI is 2.65 to 87.57 Euros/person/day. The estimated cost of treatment is 1.71 to 470.49 Euros/person/day [5]. It is important, but difficult, to monitor and manage PI.

Human adipose-derived stem cells (ASCs) are abundant and easily collected at a relatively low cost and are an option for PI repair and tissue reconstruction. ASCs secrete various factors that promote the growth of fibroblasts and epidermal, vascular endothelial, and nerve cells. They also secrete immunoregulatory factors, chemokines including interleukins, and monocyte chemotactic proteins [6]. Local transplantation of ASCs promotes PI healing in animal models [7], but ASC suspensions without extracellular matrix (ECM) components stimulate immune responses that shorten cell survival [8]. It appears that transplantation of ASC suspensions alone is not sufficient to enhance healing [9].

Platelet-rich plasma (PRP) contains a high concentration of platelets that release growth factors and cytokines including platelet-derived growth factor (PDGF), basic fibroblast growth factor (bFGF), vascular endothelial growth factor (VEGF), insulin-like growth factor-1 (IGF-10), and transforming growth factor-*β* (TGF-*β*) and others that are involved in angiogenesis, tissue repair, and inflammatory activity. As the growth factors and cytokines released by platelets facilitate wound healing, PRP is currently used to prevent and treat PI [10–12], but some PRP proteins are cleared in a short time and lead to loss of activity. Evidence from the few published randomized controlled trials is not sufficient to support PRP alone for treating chronic wounds [13].

The synergistic effects of PRP and human ASCs on the promotion of healing have been shown by enhanced vascularization of full-thickness wounds in a porcine model [9]. Moreover, PRP combined with ASCs might be beneficial for treating radiogenic wounds [14]. In addition, an in vitro study has demonstrated that PRP can facilitate neurogenic differentiation of ASCs [15]. Although the pathogenesis of PI is complex and involves angiogenesis and neurogenesis disorders, we hypothesize that local transplantation of PRP combined with ASCs may play a synergistic effect on promoting PI wound healing by increasing angiogenesis and neurogenesis. Therefore, we established a mouse PI model described by Strong et al. [7], and mice with local transplantation of ASCs or PRP are controls. To our knowledge, this is the first report to investigate the effectiveness and mechanisms of PRP combined with ASCs to treat PIs.

## 2. Materials and Methods

### 2.1. Ethical Approval

The Ethics Committee of the Affiliated Hospital of Zunyi Medical University approved the protocol, and all procedures involving animals were approved by the institutional Animal Care and Use Committee and conducted following the guidelines of the China National Health and Medical Research Council. All human participants gave signed informed consent.

### 2.2. Isolation and Culture of ASCs

Subcutaneous adipose tissue was harvested from plastic surgery inpatients at the Affiliated Hospital of Zunyi Medical University, and ASCs were isolated as previously described [16]. Briefly, the adipose tissue was minced, washed, and digested with 0.075% collagenase type I (Sigma, St. Louis, MO, USA) for 45 min in a shaker incubator at 37°C. Adipocytes and undigested connective tissue were discarded after centrifugation at 800 × *g* for 5 minutes. The pellets were resuspended in phosphate-buffered saline (PBS, Gibco, Carlsbad, CA, USA) and filtered through 200 *μ*m mesh screens before centrifugation at 800 × *g* for 5 min to harvest the stromal-vascular fraction. The harvested cells were cultured at 37°C and 5% CO_2_ in complete Dulbecco's modified Eagle's medium with 10% fetal bovine serum and 1% penicillin and streptomycin (all from Gibco). The ASCs were subcultured when they reached 80% confluence; passage three cells were used in the experimental procedures.

### 2.3. Flow Cytometry of ASCs

A flow cytometric assay of cell surface marker expression was conducted in 1 × 10^6^ ASCs that had been stained with anti-CD90, anti-CD73, anti-CD105, anti-CD34, anti-CD11b, anti-CD19, anti-CD45, and anti-HLADR antibodies (1 mg/ml; Abcam, Cambridge, UK) and suspended in PBS. The samples were incubated for 30 minutes at room temperature, washed with PBS, and then analyzed with a MoFlo XDP flow cytometer (Beckman Coulter, Brea, CA) and Kaluza software (Beckman Coulter).

### 2.4. Induction of ASC Differentiation

In vitro differentiation was performed as previously described [17]. Adipogenesis and osteogenesis were assayed on day 21 by 1% oil red O and alizarin red S staining. Chondrogenesis was assayed on day 28 by 1% alizarin blue staining. The assays were performed in triplicate.

### 2.5. Preparation of PRP

Human PRP was prepared as previously described [14, 18]. Briefly, peripheral blood was collected from healthy volunteers into vacuum tubes containing sodium citrate anticoagulant. The sample was centrifuged at 900 g/min for 5 minutes at room temperature. The whole blood was divided into three layers: the upper layer was the supernatant, the lower layer was the red blood cells, and the middle layer was the platelet layer. The platelet layer was centrifuged at 1500 g/min for 15 min to give an upper platelet-poor and lower platelet-rich layer. After performing a platelet count of the lower layer, 10% calcium gluconate was added to form a 1 : 10/*v* : *v* suspension. Platelets were activated for 1 h, centrifuged at 800 g/min for 5 minutes, and passed through a 0.22 *μ*m filter for use.

### 2.6. Establishment of the PI Model

Healthy female wild-type C57BL/6 mice were purchased from Chongqing Tengxin Bill Animals Sales Co., Ltd. (license no. SCXK (Yu) 2012-0005) at 6–8 weeks of age. The mice were allowed to acclimate for at least 1 week before inducing PI by magnet application as previously described [7]. Briefly, mice were anesthetized by intraperitoneal injection of 0.1 ml/10 g chloral hydrate, the concentration and dose that resulted in the fewest adverse effects. To further decrease the adverse effects of chloral hydrate, a small amount of ketamine (100 mg/kg) was added as an adjuvant. The dorsal hair was shaved, and the skin was gently pulled up and placed between two 12 mm diameter 5 mm thick neodymium magnets (Shanghai Qi Hao Electrical Co., Ltd., China) for 12 h. The magnets were removed after a 12 h ischemia-reperfusion cycle. Mice were exposed to two IR cycles, resulting in two wounds per mouse after 2 days. Histological evaluation of the wounds confirmed the establishment of the PI model.

### 2.7. Intervention in the Experimental Model

The model mice were randomly allocated to four groups of 20 mice each: ASCs combined with PRP, ASCs alone, PRP alone, and a control group. ASCs were transfected with lentivirus expressing green fluorescent protein (GFP). To perform the study treatments, the wounds of model mice were injected subcutaneously with 1.0 × 10^6^ GFP^+^ ASCs suspended in 100 *μ*l PRP or in sterile PBS or with 100 *μ*l PRP or sterile PBS using 27-gauge needles and 1 ml Luer-Lok syringes. Injections were done under anesthesia. Each mouse received a total of 200 *μ*l (100 *μ*l per wound; two wounds per mouse). The injection sites were sealed with an antibacterial protective film (3M, St. Paul, MN). The wounds were photographed on days 1, 5, 9, 13, 17, and 21 after injection. A steel ruler was included in the picture. Wound size was estimated by ImageJ software (Rawak Software, Inc., Germany). Wound healing was recorded as a percentage: (initial wound size − residual wound size)/initial wound size × 100.

### 2.8. Histological Evaluation

One-third of the mice were sacrificed on days 5, 11, and 21 after injection, and the wound skin was harvested and fixed in 10% neutral buffered formalin (Sigma-Aldrich) for at least 48 hours, embedded in paraffin and sectioned at 4 *μ*m, and mounted on glass slides. The sections were deparaffinized, rehydrated in HistoChoice and a graded ethanol series, and stained with hematoxylin and eosin (HE) or Masson's trichrome stain according to the manufacturer's instructions. Skin sections stained with HE were evaluated for inflammatory cell infiltration using ImageJ software. Sections stained with Masson's trichrome were assessed for the percentage of collagen. An ImageJ algorithm was used to count the number of blue pixels and calculate the percentage of the total number of pixels per skin section.

### 2.9. Immunohistochemical Staining

Angiogenesis and neurogenesis were evaluated by immunohistochemical staining of the vascular endothelium with anti-CD31 (1 : 200; Abcam) and Schwann cells with anti-S100 (1 : 150; Abcam). Specimens were pretreated for antigen retrieval by heating followed by blocking with 1% bovine serum albumin (Sigma) and incubated with a primary antibody at 4°C overnight. Specimens were washed with PBS, incubated with a secondary antibody (1 : 250; Abcam) for 30 min, stained with diaminobenzidine (Invitrogen) and counterstained with hematoxylin, dehydrated, and cover slipped. Neovascularization of serial sections of specimens was evaluated by light microscopy at high magnification. The percentage of S100-positive cells was determined by ImageJ software, and five high-power fields per sample were included in the analysis.

### 2.10. Immunofluorescence Staining

Frozen sections were obtained from the wound periphery on day 21 after ASC injection. Samples were treated with a frozen embedding agent (Invitrogen). Frozen sections were made and washed with PBS. Nuclei were stained for 5 minutes with diamidino-phenylindole (DAPI, 1 : 1000; Sigma) and washed with PBS. After drying, sections were observed with an inverted fluorescence microscope. With blue light excitation, ASCs appeared green and DAPI appeared blue.

### 2.11. Statistical Analysis

The results were reported as means ± standard deviation after Shapiro–Wilk testing for normal distribution. Student's *t*-test was used to compare between-group differences at a single time. One-way analysis of variance was used to compare the groups at all times. *P* values < 0.05 were considered statistically significant.

## 3. Results

### 3.1. Cultivation and Identification of ASCs

**Figure 1:**
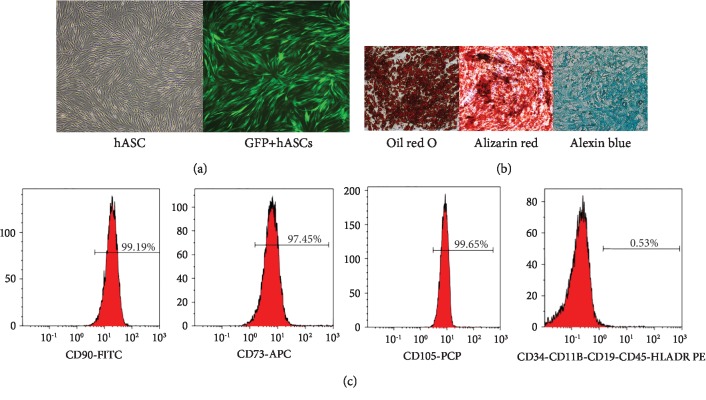
ASCs from human subcutaneous adipose tissue had characteristics of mesenchymal stem cells. Representative phase-contrast micrographs of ASCs show fibroblast morphology. The percentage of GFP-transfected cells was high; the morphology did not change significantly (a). ASCs differentiated into adipocyte-, osteoblast-, and cartilage-like cells (×100 magnification) (b). Flow cytometry of ASCs (c). ASCs: adipose-derived stem cells.

ASCs isolated from the subcutaneous adipose tissue of inpatients were characterized by microscopy and the cell surface marker profile. ASCs had a fibroblast-like morphology and expressed GFP on fluorescence imaging (Figure 1). Flow cytometric analysis revealed 95% expression of MSC-specific CD90, CD73, and CD105 and hematopoietic-specific CD34, CD11b, CD19, CD45, and HLADR markers (Figure 1). After incubation of ASCs in adipogenic induction medium for 3 weeks, round lipid droplets of various sizes were observed. Red granules visible after oil red staining confirmed the differentiation of ASCs into adipocytes. Osteoblast differentiation was indicated by alizarin red staining of calcium salt nodules. After 4 weeks of induction culture, alizarin blue staining revealed large amounts of acid mucopolysaccharide in cartilage tissue, indicating that the cultured ASCs had differentiated into chondrocytes (Figure 1).

### 3.2. Confirmation of the PI Model

**Figure 2:**
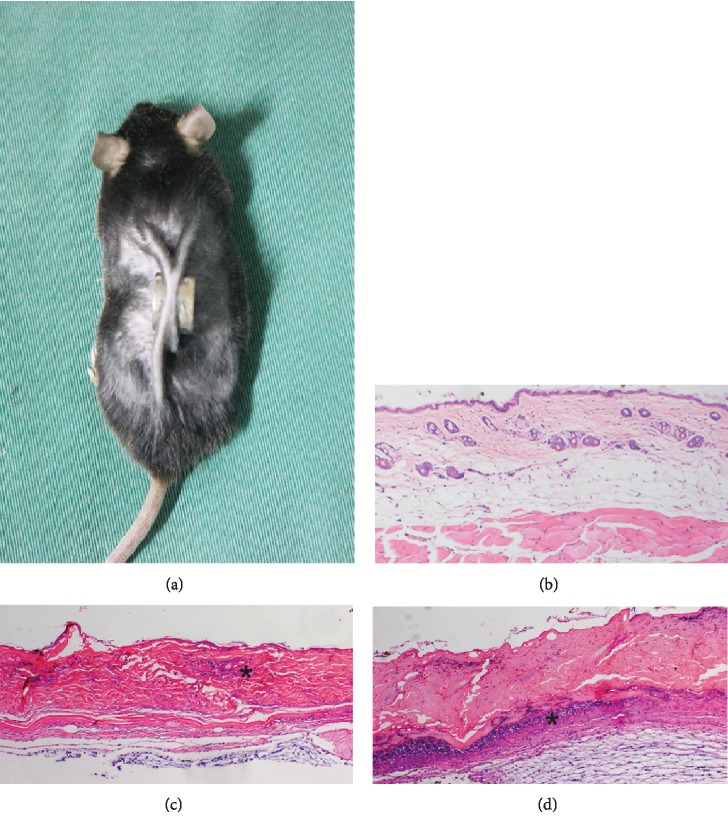
Animal model of pressure injury (PI) (a). Normal skin structure (b). On day 1 after the establishment of the PI model, skin thinning, loss of the normal skin structure, and inflammatory cell infiltration (∗) are visible (c). On day 5, the epidermis was exfoliated, degeneration and necrosis of subcutaneous tissue are visible, and infiltration of inflammatory cells is extensive (∗) (d). ×100 magnification.

Histological evaluation of HE-stained tissue from model mice on day 1 after modeling found that the normal structure of the epidermis and subcutaneous fat had disappeared and that a small amount of inflammatory cell infiltration was present in the dermis. On day 5, degeneration and necrosis of the dermis had occurred and infiltration of inflammatory cells was extensive, confirming successful establishment of the PI model (Figure 2).

### 3.3. PRP Combined with ASC Treatment Enhanced PI Healing

**Figure 3:**
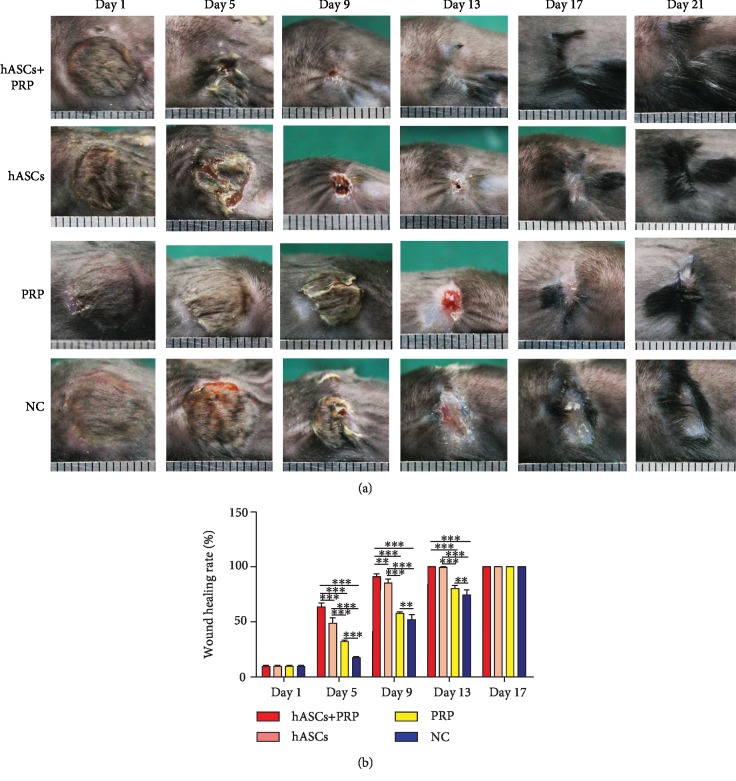
PRP combined with ASCs, ASCs, or PRP alone was injected subdermally at the wound margins. Healing of pressure wounds was monitored. Representative images of wound healing at increasing times after local transplantation of PRP combined with ASCs, ASCs, or PRP (a). Histogram of wound healing over 17 days showing significant differences in the rate of healing (b). ^∗∗^*P* < 0.01, ^∗∗∗^*P* < 0.001. ASCs: human adipose-derived stem cells; PRP: platelet-rich plasma.

The efficacy of ASCs in PI wound healing was evaluated in model mice at 6–8 weeks of age. A total of 1.0 × 10^6^ ASCs were injected into the base of the wounds and assessed over 21 days. Delivery of PRP and ASCs to the wound site accelerated wound healing. As early as day 5, mice treated with PRP and ASCs had higher percentages of wound healing compared with mice treated with ASCs, PRP, or PBS alone. The trend of acceleration of wound healing by PRP combined with ASC continued until wound closure was completed on day 13. The percentages of wound healing on days 5, 9, and 13 were higher in mice treated with ASCs than in those treated with PRP (Figure 3).

### 3.4. PRP Combined with ASCs Suppressed Periwound Inflammation and Promoted Regeneration of Skin Appendages

**Figure 4:**
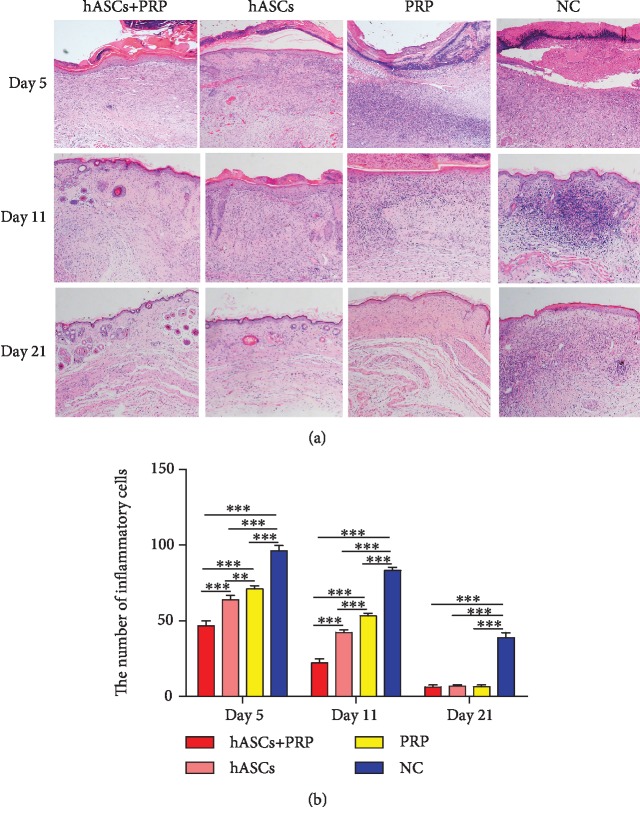
PRP combined with ASCs, ASCs, or PRP alone suppressed the inflammatory response to wounding. Tissue histology shows that inflammatory cell infiltration in the dermis and subcutaneous layers on days 5 and 11 was less in tissue from ASC plus PRP-treated mice than from controls. The infiltration of inflammatory cells was the least extensive in ASC plus PRP-treated mice (a, b). ×100. ^∗∗^*P* < 0.01, ^∗∗∗^*P* < 0.001.

HE staining of PI-wounded skin on days 5 and 11 revealed that PRP combined with ASCs significantly reduced the infiltration of inflammatory cells in the dermis compared with ASCs, PRP, or PBS alone. Infiltration was also less extensive in ASC-treated mice than it was in mice treated with PRP or PBS. Inflammation was the most severe in PBS-treated control mice. Skin appendages had regenerated by day 11 in mice in the ASC plus PRP group, and none were observed in mice in the ASC, PRP, and PBS-control groups. The wounds in mice treated with ASC combined with PRP had nearly normal skin structure; many adnexal structures had regenerated. The epidermis was tightly connected to the dermis, and skin appendages had regenerated in the dermis of the ASC-treated mice. Skin appendages were not observed in the mice in the PRP and PBS-control groups (Figure 4).

### 3.5. PRP Combined with ASCs Increased Periwound Collagen Deposition

**Figure 5:**
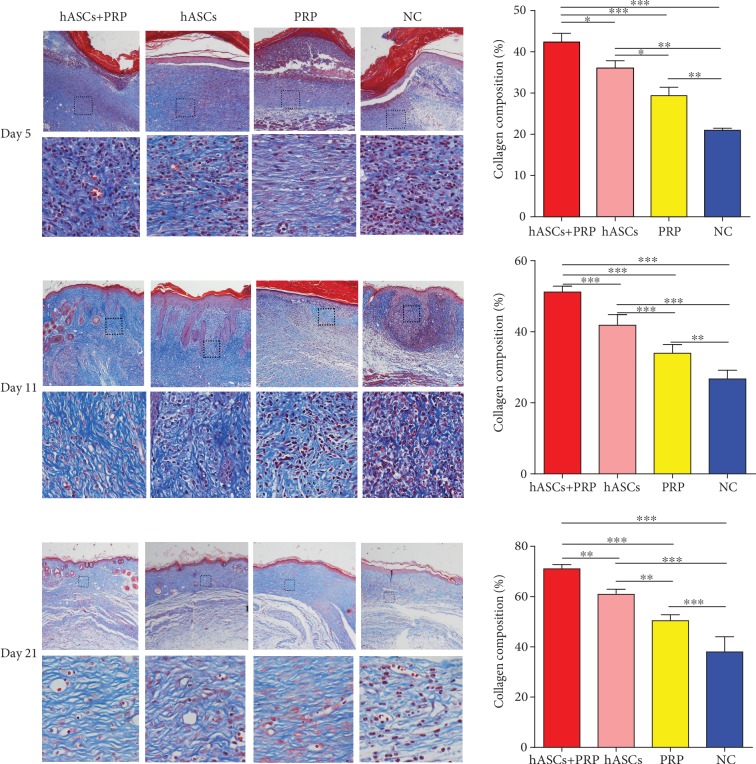
Collagen accumulation in PI wounds on days 5, 11, and 21. Masson staining shows that collagen deposition in the dermis was increased in ASC plus PRP-treated mice compared with the ASC group, PRP group, and controls. Collagen synthesis in wound skin. ×100 and ×200. ^∗^*P* < 0.05, ^∗∗^*P* < 0.01, and ^∗∗∗^*P* < 0.001.

Masson staining of wound tissue collected on days 5, 11, and 21 revealed more extensive collagen deposition in the dermis of mice treated with PRP and ASCs compared with those treated with ASCs, PRP, or PBS alone. Similarly, more collagen deposition in the dermis of mice treated with ASCs was revealed compared with that of mice treated with PRP or PBS, and more collagen deposition in the dermis of mice treated with PRP was revealed compared with that of mice treated with PBS on days 5, 11, and 21 (Figure 5).

### 3.6. PRP Combined with ASC Treatment Increased the Periwound Angiogenesis

**Figure 6:**
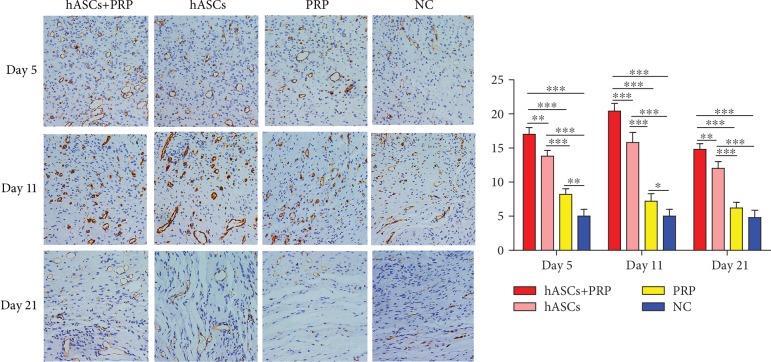
Effects of ASCs or PRP on wound neovascularization on days 5, 11, and 21 after cell or PRP transplantation. Immunohistochemical staining to reveal CD31 expression on days 5, 11, and 21 found more new blood vessels in tissue from ASC plus PRP-treated mice than from mice in the other groups. In all groups, fewer new blood vessels were seen on day 21 than on days 5 and 11. Formation of new vessels in PRP-treated and control mice on day 21 was not significantly different. ×200. ^∗^*P* < 0.05, ^∗∗^*P* < 0.01, and ^∗∗∗^*P* < 0.001.

An immunohistochemical assay of CD31 expression in vascular endothelial cells collected on days 5, 11, and 21 found more new blood vessels in mice treated with PRP and ASCs than in mice treated with ASCs, PRP, or PBS alone. More new blood vessels were found in the dermis of mice treated with ASCs compared with those treated with PRP or PBS. On days 5 and 11, mice treated with PRP had more new blood vessels than those treated with PBS, but the difference on day 21 was no longer significant (Figure 6).

### 3.7. PRP Combined with ASCs Increased Periwound Neuroregeneration

**Figure 7:**
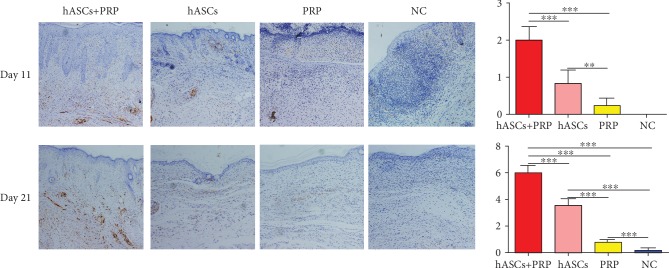
Effects of ASCs or PRP on wound neuroregeneration on days 11 and 21. Immunohistochemical staining of S100 in nerve cells in the dermis was increased more by ASCs plus PRP than by the other treatments. ×200. ^∗∗^*P* < 0.01, ^∗∗∗^*P* < 0.001.

An immunohistochemical assay of S100 expression in Schwann cells collected on day 11 found more new Schwann cells in mice treated with PRP and ASCs than in those treated with ASCs or PRP alone. S100 expression was absent in PBS-control mice. On day 21, mice treated with PRP and ASCs had more new Schwann cells than those treated with ASCs or PRP alone or PBS. More new Schwann cells were seen in the dermis of mice treated with ASCs compared with those treated with PRP or PBS alone. Mice treated with PRP had more Schwann cells than those treated with PBS (Figure 7).

### 3.8. Periwound Homing of ASCs In Vivo

**Figure 8:**
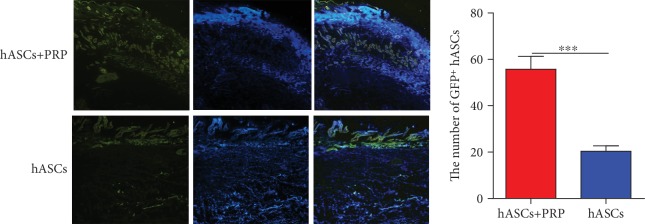
ASCs tracked in vivo in mice with PI wounds. Accumulation of GFP-labelled ASCs at the wound margin on day 21 after transplantation of ASCs and ASCs combined with PRP. ×100.

Immunofluorescence assays found that GFP-positive ASCs accumulated in the subdermal layer at the wound margins of mice treated with PRP and ASCs or ASCs alone on day 21. The accumulation was greater in mice treated with PRP and ASCs than in those treated with ASCs only (Figure 8).

## 4. Discussion

Cutaneous wound healing involves well-orchestrated regulation of inflammation, cell proliferation, remodeling, deposition of the extracellular matrix, angiogenesis, and epithelialization [19]. Unbalanced regulation of those events can result in chronic, refractory, and nonhealing wound. PI is a common type of refractory wound that involves impaired neurological function and angiogenesis [3]. Current treatment includes surgical debridement, negative pressure wound therapy, flap transplantation, hyperbaric oxygen therapy, and application of bioengineered skin substitutes [20–22]. No specific and effective treatments based on the pathophysiological mechanisms of PI are available. This study showed that local injection of PRP and ASCs, ASCs, or PRP alone promoted would healing in a mouse PI model. PRP combined with ASCs promoted faster PI healing than either ASCs or PRP alone. Histological evaluation showed faster recovery of the stratified skin structure and more extensive collagen production, angiogenesis, and neurogenesis in mice treated with PRP and ASCs than what was achieved with the other study treatments. The accumulation of GFP-labelled cells found that the preservation of ASCs was prolonged by mixing with PRP.

Stem cell therapy is a novel modality for the management of PIs and depends on precise coordination of immunomodulation, reepithelization, angiogenesis, and neurogenesis [23]. Treatment of chronic wounds with MSCs offers advantage of low immunogenicity and few ethical limitations [24, 25]. MSCs include bone marrow mesenchymal stem cells (BMSCs), ASCs, and amniotic mesenchymal stem cells. BMSCs have been shown to promote the healing of chronic wounds [26–28]. Motegi et al. reported that local transplantation of allogeneic BMSCs prevented the occurrence of pressure ulcers in a rat model of ischemia-reperfusion injury by reducing vascular damage, oxidative stress, and apoptosis [29]. Compared with BMSCs, ASCs appear to have lower immunogenicity when used for allogeneic or xenotransplantation because they lack MHC class II or costimulatory molecules [30]. For example, de la Garza-Rodea et al. reported that transplantation of human BMSCs had no significant effect on the healing of pressure ulcer wounds in immunodeficient mice [31], whilst Miranville et al. found that transplantation of ASCs promoted neovascularization in a hindlimb ischemia model in immunocompetent mice [32]. Consistent with those findings, our studies showed that local transplantation of ASCs promoted PI healing in immunocompetent mice by increasing collagen production, angiogenesis, and neuroregeneration. In addition, large numbers of ASCs are easy to obtain. A gram of adipose tissue can yield about 5000 fibroblast colony-forming units of ASCs. A milliliter of bone marrow yields only 100–1000 fibroblast colony-forming units of BMSCs making ASCs theoretically superior as a seed cell for wound healing [33, 34]. However, long-term preservation following local transplantation of ASC suspensions alone may not be ideal [8]. This study found that the number of ASCs within wounds was greatly reduced on day 21 after local transplantation.

PRP is an autologous blood product enriched in platelets, growth factors, chemokines, and cytokines. Many studies had confirmed that PRP promotes tissue regeneration, improves wound healing, and promotes blood vessel growth by secreting platelet-derived biologic mediators [13, 35–37]. Local transplantation of autologous PRP has been found to cure refractory ulcers of various causes in a group of 24 patients [38], but high-level evidence of a clear benefit for wound healing over conventional treatments in large studies is lacking [13, 39, 40]. The reason may be related to the short half-lives of the growth factors present in PRP [41]. However, fibrin networks that form in PRP provide a three-dimensional structure that can be used to make cell scaffolds. Previous studies have reported that PRP promoted the proliferation of cultured ASCs in vitro [42–44]. PRP has low immunogenicity and can be used for transplantation of allogeneic or xenogeneic cells without causing immune rejection [45]. In this study, PRP combined with ASCs was used to investigate wound healing in a mouse PI model.

We found that PRP combined with ASCs promoted PI healing more rapidly than ASCs or PRP alone. Cell tracing or accumulation of GFP-labelled ASCs found better long-term preservation with PRP plus ASCs than with ASCs alone. The results suggest that the synergistic effects of PRP plus ASCs were attributable to increased survival of ASCs and promotion of angiogenesis and neurogenesis that inhibited the pathophysiological mechanisms involved in PI. A microenvironment suitable for wound healing includes physical and chemical factors that regulate cell activity. The compositional and biological properties of PRP may provide a highly favorable environment for ASCs in which the fibrin in PRP can form a scaffold to support the attachment of ASCs and provide a nutrient-rich microenvironment to prolong the time that they remain at the transplant site [46–48]. PRP is rich in various growth factors, including PDGF-BB, FGF-2, EGF, and VEGF that might interact to promote the proliferation of hASCs through multiple signaling pathways, such as ERK1/2, PI3K/Akt, and JNK [49]. Evidence from previous studies indicates that combining PRP with ASCs is beneficial for wound healing. Plasma rich in platelet growth factors (PRGF) has been reported to maintain the biological properties of ASCs, including cell proliferation, migration, differentiation, and survival, and to prevent oxidative cell death when it is used as a preconditioning agent [50]. Another study reported that a microenvironment including an adhesive scaffold and growth factors found in PRP increased keratinocyte differentiation by ASCs [51].

Neovascularization is a key event in the healing of wounds, and in this study, it was increased by combining PRP with ASCs. It was previously reported that the presence of PRP increased the immunoreactivity and VEGF and FGF secretion by ASCs consistent with a synergistic effect on angiogenesis [52]. ASCs also exhibit epithelial cell characteristics when cultured in medium containing PRP, including high expression of the endothelial cell surface markers CD31 and VEGF. Under those conditions, secretion of hypoxia-inducible factor is increased, which activates angiogenesis and the formation of tubular structure [53]. The concentrations of growth factors that promote angiogenesis, including VEGF, TGF-*β*1, and PDGF-BB, were significantly increased by including PRP in cultures of ASCs [9]. PRP hydrogels have also been reported to increase angiogenesis by ASCs in vitro and in vivo [54].

Nerve regeneration also occurs during successful treatment of PI. In this study, the combination of PRP and ASCs increased Schwann cell regeneration and the resulting promotion of the growth and maturation of peripheral axons. Our results are consistent with those of Li et al. who found that adding PRP in the induction medium of ASCs increased the expression of nerve-specific factors including neuron-specific enolase (NSE), membrane associated protein-2 (MAP-2), growth-associated protein-43 (GAP-43), neural cell adhesion molecule (NCAM), and synapsin-1 (SYN1) [15]. The available evidence shows that PRP enhances neurogenic differentiation of ASCs and supports the potential use of hASC in nerve regeneration. Further research is needed to investigate the mechanism.

Local transplantation of ASCs or PRP alone accelerated PI healing. A large number of new vessels were observed in the PRP group on days 5 and 11, but the vascularization was significantly decreased on day 21. PRP treatment did not significantly increase angiogenesis compared with controls, possibly because the key growth proteins in PRP had short half-lives and were easily inactivated. Previous studies reported that sustained-release carriers combined with PRP significantly enhanced treatment effects compared with PRP alone [55]. The study's shortcomings include the collection of ASCs from plastic surgery inpatients and PRP from volunteers. The two were not derived from the same donor. There is also a lack of direct data from coculture of ASCs and PRP in vitro, and there is a lack of evidence of the final fate of the ASCs. In upcoming studies, we will track the long-term survival of ASCs and whether they differentiate into functional cells during wound healing.

## 5. Conclusion

PRP combined with ASCs can improve PI healing in mice by reducing inflammatory infiltration, increasing collagen deposition, promoting angiogenesis and neurogenesis, and increasing the survival time of ASCs.

## Data Availability

The data used to support the findings of this study are included within the article.
